# Supplementary material for: Narrative Messages and the Use of Emotional Appeals on Endometriosis Screening Intention: The Mediating Role of Positive Affect
Source: Int J Environ Res Public Health. 2023 Jun 23;20(13):6209. doi: 10.3390/ijerph20136209 (PMC10341228; doi:10.3390/ijerph20136209)
Supplement: Supplementary file 1 [file ijerph-20-06209-s001.zip › ijerph-2396234-supplementary.pdf]

Supplementary Materials

Section S1. Experimental Stimuli

Figure S1. Non-Narrative Condition *(Prompt: You are being asked to evaluate a health message presented to you. The message will be presented to you in the form of an* ***article****. Please take your time and read carefully all the way through to the end. Following the message, you will answer a set of questions related to what you just read. The next button will appear after* ***a couple of minutes*** *to allow you time to read the full message. Please do* ***not*** *refresh the page.*

BuzzFeed

Endometriosis: *What is it?*

This message will provide information about endometriosis.

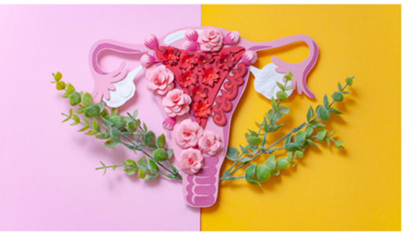

Endometriosis is a long-term disease that impacts the uterus, which is the body part responsible for your period and is where a baby develops until birth. Only those with female sex organs can have this disease. This disease happens when tissue that grows inside the uterus starts to grow outside of the uterus.

This growth of tissue is similar to normal tissue, but there is no blood flow to carry the tissue out of the body. This can lead to the tissue attaching to other body parts like the bladder. This extra tissue can result in feeling sharp and stabbing pain between the hips.

Signs of endometriosis include: pain between your hips, in your stomach, and lower back, especially during your period, heavy bleeding, pain during sex, constipation, bloating and diarrhea, as well as potential issues with ability to get pregnant and have babies.

Since this disease happens around a woman's uterus, they may experience bleeding, even when they are not on their periods. Another possible sign of endometriosis can be longer periods. People may have periods for over 1-2 weeks if they have endometriosis.

1 in 10 women between ages 12-51 years old have endometriosis. You or someone you know may have endometriosis. What are some of the potential factors that can make it more likely for one to get endometriosis? It may include having your first period at a young age, having higher levels of hormones in the body, and having one or more female family members (mother, aunt, or sister) with endometriosis, since this disease can be passed down through genetics.

A major issue is that women are not getting screened or talking to their doctor about endometriosis. Women are often told that they have other health problems like cysts on one's ovaries, sexually transmitted diseases, constipation, and even appendix issues when they actually have endometriosis.

You can find out if you have endometriosis by talking with a doctor or gynecologist. First, the doctor will talk to you about your family medical history. If the doctor believes you have endometriosis, they will do a pelvic exam to be sure of this.You can also find out if you have endometriosis through other screening options such as ultrasounds or an X-ray.

Treating endometriosis can include taking birth control that can balance out hormone levels. Different types of birth control have different hormone levels which can make periods less painful.

Other options can be removing the tissue through surgery. This helps stop the growth of the unusual tissue. However, a downside of this surgery would be not being able to have babies if that is something you wanted to do.

If you believe you may have endometriosis, the only way to know is to talk to your primary care doctor or gynecologist about getting screened. If you are not sure if you are at risk of having endometriosis, talk with your doctor.

Figure S2. Fear Appeal Narrative Condition *(Prompt: You are about to read a story about Tracy. Tracy is 22 years old and suffers from endometriosis. Please take your time and read carefully all the way through to the end. Following the message, you will answer a set of questions related to what you just read. The next button will appear after* ***a couple of minutes*** *to allow you time to read the full message. Please do* ***not*** *refresh the page.)*

BuzzFeed

Tracy's Experience with Endometriosis

Tracy is a 22 year old woman explaining her endometriosis.

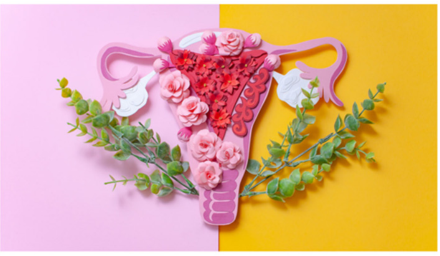

My period has caused me constant pain for as long as I can remember. Even if I wasn't menstruating, I was always experiencing sharp, stabbing pain between my hips. I couldn't function normally because of the pain I was experiencing. I allowed it to go on for so long that eventually I talked to my mom thinking she might know what was going on.

When I told her my symptoms and how long I was having them, my mother told me that she was unsure what exactly could be wrong and told me to take ibuprofen when I experience period cramps. After this, I muddled through life always feeling period pain even not on my period.

The pain was so debilitating, I was so scared of what could be wrong with me.

Just when I thought it couldn't be worse, I started to experience serious digestive issues that left me bloated and constipated. Then, it started to affect my ability to have sex with my boyfriend. The pain was so intense, there was no way we could have sex. On top of that, the pain made it difficult to do any of my regular activities.

These symptoms became so terrifying, I had to make an appointment with my gynecologist. When I asked her about my symptoms, we first had to rule out any chances of STI/STDs that could be causing this. Once we knew this wasn't what was causing my pain, the doctor informed me that my symptoms could be endometriosis.

The doctor asked if I had any family history of endometriosis, but I had never heard of the disease before my appointment. In order to determine this, she had to conduct an endometriosis screening during a pelvic exam and do an ultrasound. After she finished, the doctor informed me that I, indeed, had endometriosis.

I was very scared because I was now confronted with many unknowns. How will I control this? Will I be able to have children? Will it get worse?

The doctor informed me that many women suffer with this disease-more than 1 in 10 women have endometriosis. Most of the time, women do not seek out treatment for the disease because they believe the pain is normal. My doctor said that, if left untreated, endometriosis can lead to infertility, bladder and bowel issues, pelvic infection, and higher risk of ovarian cancer.

Even though the doctor answered my questions, I was still fearful of what endometriosis would mean for my future. My doctor was able to prescribe me low-hormone birth control to control my symptoms, but she said there is no cure for endometriosis and birth control doesn't always work for everyone. The only way to deal with it is getting a screening and getting medical treatment to control the disease before it gets worse.

I now see my gynecologist once a year in order to make sure my endometriosis does not get out of control because I could be at high risk for a number of health issues, including infertility. After I got diagnosed, I still feel scared and uncertain of my future with endometriosis. Asking your gynecologist about endometriosis screening is the only way to get answers.

Figure S3. Hope Appeal Narrative Condition *(Prompt: You are about to read a story about Tracy. Tracy is 22 years old and suffers from endometriosis. Please take your time and read carefully all the way through to the end. Following the message, you will answer a set of questions related to what you just read. The next button will appear after a* ***couple of minutes*** *to allow you time to read the full message. Please do* ***not*** *refresh the page.)*

BuzzFeed

Tracy's Experience with Endometriosis

Tracy is a 22 year old woman explaining her endometriosis.

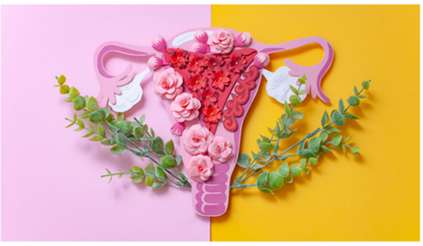

For as long as I can remember, my periods have always been intense. Everyone talks about what a pain it is to get their period, but mine seem so different. The pain from my period is so intense that some days I can't get out of bed. They would last for almost two weeks and there were times where I had to miss classes because I couldn't handle the pain. It was sharp and stabbing between my hips. When I told my mother about the pain, she disregarded it implying that I was over exaggerating, and that pain is normal during your menstrual cycle.

But this didn't feel normal.

On top of the pain, I was experiencing terrible digestive issues leaving me bloated and constipated. Now, it's becoming difficult to have sex. The pain is even worse when my boyfriend and I try to have sex. I felt hopeless. I knew I couldn't keep going on like this, so I made an appointment with a gynecologist. I asked her about what could be causing the pain and, when she ruled out things like STIs/STDs and other potential issues, she asked if there was a history of endometriosis in my family.

I never heard of this disease, but the doctor described the exact symptoms I was having. She screened me for endometriosis during my pelvic exam and suggested we do an ultrasound. After doing the ultrasound on my pelvic area, she said based on my symptoms and exam, I have endometriosis.

I was terrified when she told me this: what does this mean? Will I be able to have kids? Is it going to get worse? She explained to me that endometriosis is when the tissue that you shed during your period grows in places outside of the uterus, which causes the pain.

She said it's more common than you would think-1 in 10 women have endometriosis, but it could be more since people like me assume periods are supposed to hurt so they never get screened.

For some women it can cause issues having children, but my doctor said it will just have to be something that I monitor in the future when I do try to have kids. The doctor eased my fear by answering all my questions, making me feel hopeful I could live a normal life.

Even though the doctor said there is no cure for the disease, I was relieved when I found out hormonal birth control could help. After a few months, I felt so much better. My pain was virtually gone, I wasn't having digestive problems anymore, and I wasn't experiencing the pain during sex. While this was the best solution for me, it may not be for others, but luckily there are other options. Had I not gotten screened for endometriosis, I probably would've continued to suffer in silence.

While this process was a struggle, I know I remained resilient and strong against the pain and did the right thing by getting screened.

I felt like my pain was so normal, but *I'm glad to know that I don't have to live that way and I've been able to reduce my pain after my screening.*

Section S2. Study Questionnaire

Pre-Exposure Questions

Screening questions
Age
How old are you? (Please type in a number)

Biological Sex
What is your biological sex?
▢ Male (1)
▢ Female (2)

Previous Screening
Have you previously been screened for endometriosis?

Endometriosis Symptoms Screening
Please answer the following questions based on how often you experience these symptoms from the options “always”, “sometimes”, “never”, or “prefer not to respond”.

During your period, do you experience pelvic, abdominal, or lower back pain that limits your activities or requires medication?

In between periods, do you experience pelvic, abdominal, or lower back pain that limits your activities or requires medication?

Is sexual intercourse painful?

Does pain ever cause you to avoid intercourse?

Are bowel movements painful before or during your period?

Prior Knowledge
Have you heard about the term "endometriosis" before?
▢ Yes (1)
▢ No (0)

If yes, please describe in your own words what ‘endometriosis’ is.

Demographics
Gender
What is your current gender identity (check ALL that apply)
▢ Male (1)

▢ Female (2)
▢ Trans male/Trans man (3)
▢ Trans female/Trans woman (4)
▢ Genderqueer/Gender non-conforming (5)
▢ Different identity (please state): (6)
▢ Prefer not to answer (99)

Race
What is your race? Mark one or more boxes that apply.
▢ White or Caucasian American (1)
▢ Black or African American (2)
▢ American Indian or Alaskan Native (3)
▢ Chinese (4)
▢ Filipino (5)
▢ Asian Indian (6)
▢ Vietnamese (7)
▢ Korean (8)
▢ Japanese (9)
▢ Other Asian (10)
▢ Native Hawaiian (11)
▢ Samoan (12)
▢ Chamorro (13)
▢ Other Pacific Islander (14)
▢ Some other race (fill in) (15)

Hispanic
Are you of Hispanic, Latino, or Spanish origin?
▢ No, not of Hispanic, Latino, or Spanish origin (1)
▢ Yes, Mexican, Mexican American, Chicano (2)
▢ Yes, Puerto Rican (3)
▢ Yes, Cuban (4)
▢ Yes, another Hispanic, Latino, or Spanish origin (5)

Contemplation Ladder (Stages of Change)
Endometriosis is a condition affecting some women in which the endometrium (the tissue that lines the inside of the uterus or womb) is present outside of the uterus.

Below are some thoughts that women have about endometriosis screening. We are interested in knowing how much each of the statements below describes your thoughts about endometriosis screening. On the ladder, please rate the extent to which you agree or disagree with each

statement on a scale of *strongly disagree* (1) to *strongly agree* (5). Please read each sentence carefully before deciding.

1. I definitely plan to get screened for endometriosis in the next 30 days.
2. I definitely plan to get screened for endometriosis in the next 6 months.
3. I often think about endometriosis, but I have no plans to get screened.
4. I sometimes think about endometriosis, but I have no plans to get screened.
5. I rarely think about endometriosis, and I have no plans to get screened.
6. I never think about endometriosis, and I have no plans to get screened.
7. I have no interest in getting screened for endometriosis.

Post-Exposure Questions

Manipulation Check Questions

Please rate the following statements based on how much you agree or disagree with the statement from *strongly disagree* (1) to *strongly agree* (7).

1. The message told a story.
2. The message had a beginning, middle, and end.
3. The message showed a personal evolution of one or more characters.
4. The story in the message had a chronological order.

[Programming notes: only those who are randomly assigned to the two narrative conditions will be asked the two questions]
How did the character feel about her disease?
-She was optimistic about her prognosis
-She was scared/uncertain about her prognosis

Self-Efficacy
Please rate each statement with how strongly you agree on a scale from 1 being "*strongly disagree*" to 5 being "*strongly agree*."

If I want…

1. I can arrange transportation to get an endometriosis screening.
2. I can arrange other things in my life to have an endometriosis screening.
3. I can talk to my gynecologist about my concerns.
4. I can get an endometriosis screening even if I am worried.
5. I can get an endometriosis screening even if I don't know what to expect.
6. I can find a way to pay for an endometriosis screening.
7. I can make an appointment for an endometriosis screening.
8. I know for sure I can get an endometriosis screening if I really want to.
9. I know how to go about getting an endometriosis screening.

10. I can find a place to have an endometriosis screening.

Affective Response
Please rate the following emotions on how much you felt them while reading the message. Rate whether you experienced the emotions on a scale from 1 being "*not at all*" to 7 being "*very much*."

1	2	3	4	5	6	7
Not At All						Very Much

How well do each of the following adjectives describe your feelings while reading the story?

1. ____Happy
2. ____Cheerful
3. ____Joyful
4. ____Upbeat

Attention Check
People vary in the amount they pay attention to these kinds of surveys. Some take them seriously and read each question, whereas others go very quickly and barely read the questions at all. If you have read this question carefully, please only select *strongly disagree*.
[Program notes: randomize response option order]

- Strongly disagree (1)
- Disagree (2)
- Neither disagree nor agree (3)
- Agree (4)
- Strongly agree (5)

Behavioral Intention
Please rate the following statements with how strongly you agree with them on a scale from 1 being "*strongly disagree*" to 5 being "*strongly agree.*"

1. I want to get screened for endometriosis in the next 4 weeks.
2. I intend to get screened for endometriosis in the next 4 weeks.
3. I intend to ask my doctor about endometriosis screening.

Section S3. Mediation Figures for H3

Figure S4. *Mediation models of hope vs. fear message conditions on screening intentions through self-efficacy (a) and positive affect (b).*

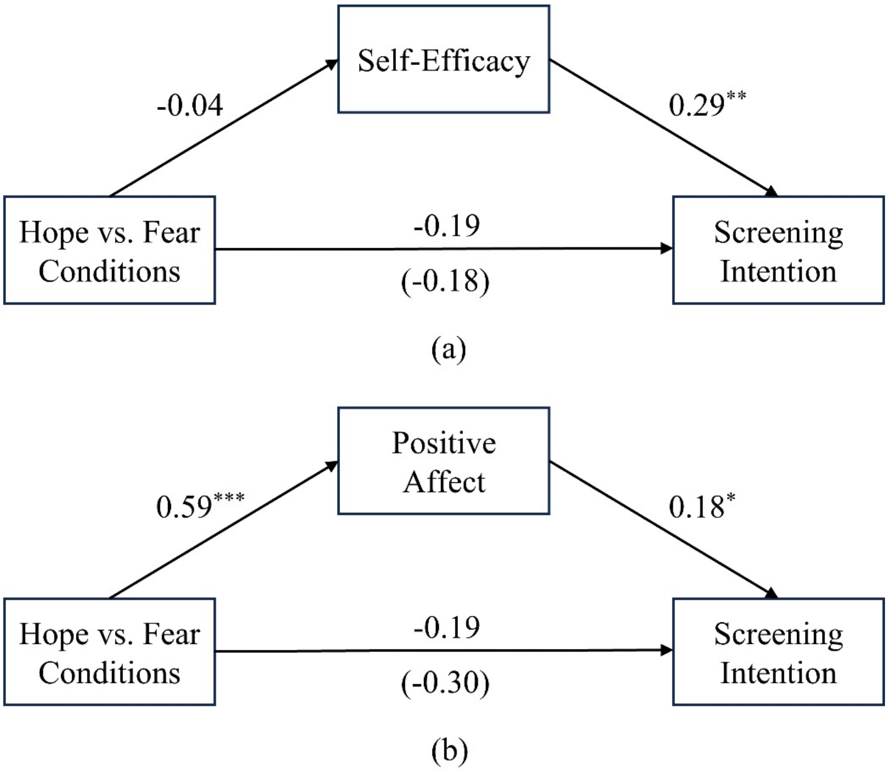

Note. Unstandardized regression coefficients are presented. The unstandardized regression coefficient between emotional appeal conditions and screening intention, controlling for self-efficacy (panel a) and positive affect (panel b), is in parentheses.
$^{*}p < .05$, $^{**}p < .01$, $^{***}p < .001$.
